# Retrospective Evaluation of Anesthetic–Analgesic Protocols in Cats with and Without Transient Myocardial Thickening Following Gonadectomy

**DOI:** 10.3390/ani16131979

**Published:** 2026-06-26

**Authors:** Claire Pollak, Laura J. Ruys

**Affiliations:** 1 AniCura Specialistisch Verwijscentrum Haaglanden, Frijdastraat 20a, 2288 EZ Rijswijk, The Netherlands; 2 Division of Small Animal Emergency and Critical Care, Department of Clinical Veterinary Science, Vetsuisse Faculty, University of Bern, 3012 Bern, Switzerland

**Keywords:** feline myocarditis, catecholamine-induced cardiomyopathy, congestive heart failure, respiratory distress

## Abstract

Transient myocardial thickening (TMT) is considered a reversible feline cardiac disease that initially mimics hypertrophic cardiomyopathy. The exact etiopathogenesis remains unknown and is likely multifactorial. General anesthesia and surgery may contribute to the development of TMT in predisposed patients due to systemic inflammation, perioperative stress, surgery itself, anesthetic management, and individual susceptibility. Additionally, inadequate analgesia could result in pain, which triggers a stress response. This stress response could stimulate endogenous catecholamine release and facilitate the development of TMT. In this retrospective study, anesthetic and analgesic protocols were compared between cats with and without TMT following gonadectomy. The study group consisted of 15 cats that presented with congestive heart failure after recent anesthesia and were later suspected of or diagnosed with TMT. The control group consisted of 300 cats without TMT after gonadectomy. All cats were considered healthy prior to gonadectomy based on physical examination; no echocardiography was performed prior to anesthesia. All cats in the TMT group were anesthetized with ketamine and an alpha-2 agonist, which was medetomidine in 14/15 (93.3%) and dexmedetomidine in 1/15 (6.7%) of the cats. In the control group, all cats were anesthetized with ketamine and dexmedetomidine. The ketamine dose was significantly higher (*p* < 0.001) in the TMT group compared to the control group, with a median dose of 6.78 mg/kg (4.52–10.34) and 4.29 mg/kg (2.08–6.45), respectively. The doses of the alpha-2 agonist and atipamezole were not significantly different. The most commonly administered non-steroidal anti-inflammatory drug was meloxicam in 83.3% of the study group cats and 98.7% of the control group cats. The dose of meloxicam was significantly lower (*p* < 0.001) in the study group compared to the control group, with median doses of 0.2 mg/kg (0–0.3) and 0.29 mg/kg (0–0.38), respectively. In the control group, 93.0% of the cats received buprenorphine, and 75.0% of males received intratesticular lidocaine. None of the cats in the study group received buprenorphine or intratesticular lidocaine during the procedure. Additionally, oxygen supplementation was significantly different (*p* < 0.017), and lack of oxygen was associated with the development of TMT. Anesthetic protocols between the two groups were significantly different. However, it is important to note that not all observed complications can solely be attributed to anesthetic or analgesic differences; effects might also be due to different perioperative circumstances.

## Simple Summary

Young cats presenting with acute respiratory distress after undergoing gonadectomy can be diagnosed with transient myocardial thickening (TMT). This is a form of feline myocarditis that can be completely resolved after a few months of treatment. The exact etiology of TMT is unknown, but stress from surgery and anesthesia could be key to its development. In our study, we compared anesthetic protocols between cats that developed TMT and cats that did not after gonadectomy. The cats that developed TMT did not receive buprenorphine or intratesticular lidocaine, had significantly lower meloxicam doses, and had higher ketamine doses. All of these may imply that insufficient analgesia during gonadectomy results in pain, which causes a stress response. This stress response increases endogenous catecholamine release, which may cause myocarditis. Ketamine itself also increases catecholamine release, which may be associated with catecholamine-induced myocarditis.

## 1. Introduction

Transient myocardial thickening (TMT) is a feline cardiac disease, first described in 2018 [1,2]. It is characterized by a hypertrophic cardiomyopathy (HCM) phenotype with reversible left ventricular wall thickening [1,2]. Some individuals have an increased left atrium/aorta ratio and a decreased contractility of the left atrium, resulting in left-sided congestive heart failure (CHF) and cardiogenic shock [1,2]. Most cats have increased cardiac troponin I (cTnI) concentrations, which indicates myocardial injury and therefore myocarditis [1,2,3,4]. The exact etiopathogenesis of TMT remains unknown, although TMT is hypothesized to be a subtype of feline myocarditis. It can be triggered via similar mechanisms [1,2]. In humans, Takotsubo cardiomyopathy (TC) is a stress- or severe illness-induced ischemic cardiomyopathy which causes decreased myocardial contractility due to increased catecholamine levels [5,6]. Similarly, stress and severe illness may induce TMT [1]. It is believed that non-ischemic damage to cardiomyocytes results in inflammation of the heart muscle, which characterizes acute myocarditis [2,7]. This inflammatory response causes transient interstitial edema, resulting in increased myocardial mass and myocardial dysfunction [2,8,9,10]. Generally, TMT and TC are known to have excellent long-term prognosis due to their reversible nature [1,2,8,9,10]. 

Transient myocardial thickening typically occurs in young cats with a median age of 2–3 years [1,2] and can be infectious or non-infectious in origin [1,2,11]. A few infectious agents have been identified as associated with the development of TMT, including *Bartonella henselae* and *Toxoplasma gondii* [12,13,14,15]. *Streptococcus canis*, feline immunodeficiency virus, feline panleukopenia virus, feline coronavirus, *Sarcocystis felis*, and *Hepatozoon silvestris* have been associated with feline myocarditis [16,17,18,19,20,21]. 

Another possible contributing factor is the presence of an antecedent event within 14 days of clinical signs developing; examples include general anesthesia, surgery, a traumatic incident, drug reaction, vaccination, bite wounds, thermal burn injury, and septicemia [1,2,4,22,23,24]. An antecedent event was described in 33.3 to 71% of cases, with general anesthesia being the most frequent event (46.7–77.8%) [1,2]. The anesthetic protocols were not mentioned in these cases. Since most of the case reports or case series focus on infectious causes of TMT, information about anesthetic drugs is lacking [23]. Only one case report mentioned the medication used prior to the development of clinical signs of CHF [23]. The cat in that case was anesthetized for ovariohysterectomy using medetomidine, methadone, and alfaxalone. Anesthesia was further maintained with isoflurane. Several hypotheses for the development of TMT in this cat were discussed, including stress-induced catecholamine release, drug hypersensitivity or a delayed drug reaction, and septicemia [7,23,24].

The following criteria for TMT have been established by Romito et al. (2023) [2]: (1) an initial echocardiographic examination showing end-diastolic thickness of the interventricular septum and/or a left ventricular free wall ≥ 6 mm; (2) a concurrent demonstration of elevated circulating cTnI (>0.2 ng/mL); (3) an echocardiographic follow-up demonstrating a subsequent reduction in both the left ventricular free wall and/or the end-diastolic thickness of the interventricular septum <5.5 mm; and (4) a concurrent reduction in cTnI compared to the first examination.

The objective of this retrospective study is to identify the anesthetic–analgesic protocols used for cats with and without TMT following gonadectomy. The purpose is to determine whether there is a significant difference in the agents and doses administered, and to determine possible confounders. It was hypothesized that insufficient analgesia could be a trigger for the development of TMT.

Since not only general anesthesia but also preoperative handling and surgery could trigger postoperative cardiac complications, the term “procedure” will be considered throughout the manuscript to accommodate these factors.

The exact distinction between TMT and feline myocarditis is unclear; as mentioned above, TMT is hypothesized to be a subtype of myocarditis. As TMT has only recently been described, a limited number of articles describe TMT, and most refer to myocarditis. In order to improve readability and understanding, it was decided to use the term “TMT” when discussing either term throughout the manuscript.

## 2. Materials and Methods

### 2.1. Study Group

For this retrospective study, data were collected by a single investigator by searching for TMT, transient myocardial thickening, and troponin in the private clinic’s medical records database. A total of 51 client-owned cats were suspected of or diagnosed with TMT between January 2019 and February 2025 following the criteria set by Romito et al. (2023) [2]. Inclusion criteria were general anesthesia for gonadectomy within 14 days prior to the development of congestive heart failure due to HCM phenotype without prior symptoms of cardiac disease and significantly elevated cTnI. Based on physical examination and cardiac auscultation, the cats were considered American Society of Anesthesiologists I (ASA I). As routine preoperative cardiologic screening is uncommon for gonadectomy, none of the cats had echocardiography prior to the surgery. Twenty-four cats were excluded because they had no history of prior anesthesia, and four additional cats were excluded because they underwent a surgery other than gonadectomy.

Exclusion criteria were evidence of congenital heart diseases, underlying diseases such as pneumonia or gastrointestinal signs, euthanasia before diagnostics or therapy could be initiated, and incomplete anesthetic records. Postoperatively, a few cats were diagnosed with congenital heart disease via echocardiography; these cats were also considered ASA I prior to the gonadectomy but were excluded from the study. This resulted in the exclusion of 6 cats due to incomplete medical records, one due to underlying congenital heart disease, and one due to concurrent pneumonia.

Fifteen cats remained, all of which underwent general anesthesia for gonadectomy at a young age (<24 months) between September 2021 and February 2025 and were considered healthy prior to the procedure. The cats underwent gonadectomy at 13 different veterinary clinics, all of which were referring first-opinion veterinary practices.

The collected variables included breed, age, sex, body weight, anesthetic protocol including anesthetic agents, oxygen therapy, monitoring, and postoperative NSAID administration, and the development of TMT. If data about oxygen supplementation and capnography were missing in the anesthetic records, they were considered absent. No other values were missing among the included observations. The analysis did not account for potential confounders, including clinician-related factors such as preoperative handling, surgical experience, tissue trauma, duration of anesthesia, clinic-related factors such as perioperative anesthetic monitoring and management, available equipment, and postoperative recovery, and patient-related factors such as occult cardiac disease, susceptibility to stress, and housing conditions. Given the limited number of cases, multivariable adjustment for potential confounders was performed but should be interpreted with caution. Potential clustering by clinic or veterinarian was not accounted for because of the limited number of TMT cases.

### 2.2. Control Group

The control group consisted of 300 client-owned cats that underwent gonadectomy between January 2021 and November 2023. The cats were retrospectively identified by a single investigator from medical records, searching for sterilization or castration in the private clinic dataset. Gonadectomy was conducted at four private primary veterinary practices by 14 different veterinarians. These referring primary veterinary practices are located in the same geographic region as those of the study group. The operating experience ranged from 1 month to 12 years at the time of the gonadectomy. Eight months were randomly selected between January 2021 and November 2023 to obtain a representative sample. All cats encountered during this study period were eligible for inclusion, except those meeting predefined exclusion criteria.

Cats were excluded from the control group when medical records were incomplete and missing the following: recent body weight (<5 days before the procedure), date of birth, medication doses, and follow-up. In order to compare the anesthetic protocols between the control and the TMT group, cats with an underlying disease, such as pyometra, were excluded from the study since these diseases could affect the clinician’s choice of medication doses. To optimally compare the two groups, a maximum age of 24 months was chosen for the control group since no cats in the study group were older than 24 months. A total of 334 records were screened: 18 cats were excluded because of incomplete anesthetic records missing crucial data about anesthetic doses, eleven cats were excluded because of old age, and five cats were excluded because of an underlying disease. No formal sample size calculation was performed because all eligible cases were included. A higher control-to-case ratio (1:20) was used due to the limited number of available cases, to improve statistical efficiency and the precision of estimates.

The cats in the control group were considered ASA I based on clinical examination prior to the surgery. None had a presenting heart murmur, arrhythmia, signs of respiratory distress, or exercise fatigue; therefore, none of the cats underwent echocardiography prior to the surgery.

Potential sources of bias include heterogeneity of anesthetic and surgical protocols, perioperative management and monitoring, and postoperative care. The different anesthetic protocols may also act as a confounding factor.

### 2.3. Statistical Analysis

Analysis was undertaken in Minitab 22 (Minitab, LLC: State College, PA, USA) and R 4.5.1 (R Foundation for Computing, Vienna, Austria); significance was taken as *p* < 0.05. Continuous variables were compared between the control and study group using tie-adjusted Mann–Whitney tests. Categorical variables were compared between the same two groups using a chi-square test, or Fisher’s exact test, where expected cell counts were low. Continuous data between survivors and non-survivors of the study group were compared using Mann–Whitney tests adjusted for ties. Variables generating separation between control and study groups at *p* < 0.05 were included in a multiple Firth binary logistic regression using the R routine Logistf. Considering the number of candidate variables and the limited number of TMT cats, the results of this regression analysis should be treated with caution.

## 3. Results

### 3.1. Study Group

#### 3.1.1. Clinical Signs at Presentation

A total of 15 cats met the inclusion criteria and were included in the study group.

All 15 cats developed respiratory signs due to CHF within two weeks after general anesthesia for gonadectomy. All were treated at the referral center; 2/15 (13.3%) were initially treated at the referring veterinarian and subsequently referred for further investigations. The median time between general anesthesia and the development of respiratory signs was 5 days (range 1–14).

Nine cats were female (60.0%) and 6/15 (40.0%) were male. Six cats (40.0%) were Domestic Shorthair, 5/15 (33.3%) were British Shorthair, 2/15 (13.3%) were British Longhair, 1/15 (6.7%) was Persian, and 1/15 (6.7%) was a mixed-breed Maine Coon. The median age of the cats was 222 days (range 174–735). The median weight was 3.29 kg (range 2.15–4.42). 

All cats had a physical examination prior to the anesthesia and were considered ASA I. General anesthesia was achieved via intramuscular injections. Not all first-line clinics could provide information about proper anesthetic monitoring and/or surgical handling. Five female cats (33.3%) received oxygen while intubated; however, only two were monitored via capnography and none received isoflurane. None of the male cats received oxygen during gonadectomy. Duration of anesthesia or operator experience was not reported for any of the cats.

None of the cats in the study group underwent serological testing for *Toxoplasma gondii*, *Bartonella henselae*, or feline immunodeficiency virus.

At presentation in our clinic, all cats were in respiratory distress. The clinical exam identified 7/15 cats (46.7%) as lethargic, bradycardic, and hypothermic, suggesting cardiogenic shock. All cats had a systolic ≤3/6 parasternal heart murmur, 9/15 cats (60.0%) had a weak pulse, and 2/15 (13.3%) had pale mucous membranes. All cats had an increased respiratory effort, an abdominal respiratory pattern, and increased lung sounds. A point-of-care ultrasound performed in 13/15 cats (86.7%) showed a significant number of B-lines and an increased left atrium/aorta ratio, suggesting CHF. Eleven cats (73.3%) received thoracic radiographs, which showed signs comparable with CHF: cardiomegaly, alveolar–interstitial patterns compatible with lung edema, and enlarged pulmonary veins. Echocardiography was either performed by a cardiologist (Dip ECVIM-CA Cardiology), a radiologist (Dip ECVDI), or a resident in diagnostic imaging in all cats. Additionally, 5/15 cats (30.0%) had pericardial effusion, 1/15 (6.7%) had pleural effusion, and 1/15 (6.7%) had spontaneous echocardiographic contrast.

#### 3.1.2. Diagnosis of TMT

Diagnosis of TMT was based on the development of CHF after general anesthesia for gonadectomy. Both general anesthesia and surgery could have contributed to the development of TMT. Cats were considered to have TMT when they had an increased cTnI level at presentation, an increased left atrium/aorta ratio, and increased left ventricle wall thickness (LVWT) compatible with an HCM phenotype [1,2]. Overall, a cTnI greater than 0.16 ng/mL is considered elevated and suspicious of myocarditis and/or TMT [25]. An initial elevation of LVWT (LVWT ≥ 6 mm) with a gradual reduction in LVWT (<5.5 mm) over time and a final resolution of CHF is considered the definition of TMT (1). Not all cats in the current study achieved the latter due to euthanasia. See Table 1 for information about each cat including anesthetic agents, cTnI concentration, and echocardiographic changes.

#### 3.1.3. Laboratory Data

Standardized bloodwork was not performed. Four cats (26.7%) developed hypokalemia (median: 2.9 mEq/mL; range: 2.7–3.0; reference interval: 3.5–5.8) during the hospitalization period and were subsequently treated with supplementary potassium chloride. N-terminal prohormone of the brain natriuretic peptide was tested in five cats (30.0%) and was abnormal in all of them (NT-proBNP IDEXX Laboratories, Westbrook, ME, USA). The median creatinine value was 124.5 µmol/L (range: 67–208; reference interval: 71–212) and was measured between two and four days after initiation of diuretic therapy in six cats.

All cats were tested for cardiac troponin I in the acute phase with a median of 9.09 ng/mL (range 1–47.38; reference interval: 0–0.06). 

#### 3.1.4. Treatment

Prior to hospitalization, a total of nine cats (60.0%) received butorphanol as mild sedation. Treatment for all cats with CHF included furosemide therapy. Ten cats received clopidogrel (66.7%), three received pimobendan (20.0%), two cats received atenolol (16.7%), and one cat received torsemide (6.7%). Two cats were treated with additional anticoagulant drugs: one with rivaroxaban and one with low-molecular-weight heparin.

#### 3.1.5. Anesthetic Records

For the TMT group, general anesthesia included ketamine and an alpha-2 agonist for all cats. Of these, 14 (93.3%) received medetomidine, and one cat (6.7%) received dexmedetomidine. The median ketamine dose was 6.78 mg/kg (4.52–10.34), the median medetomidine dose was 70 µg/kg (10–100), and the dexmedetomidine dose was 20 µg/kg. Twelve cats (80.0%) that received an alpha-2 agonist were antagonized with atipamezole at 0.15 mg/kg (0.07–0.26).

Twelve cats (80.0%) received non-steroidal anti-inflammatory agents during or after surgery, 10/12 cats (83.3%) received meloxicam at 0.2 mg/kg (0.18–0.3), and 2/12 (16.7%) received tolfenamic acid at 4 mg/kg. None of the cats in the study group received additional analgesia. Three cats (20.0%) did not receive any NSAID; of these, two were male. Nine cats (60%) received postoperative NSAIDs for continued pain medication at home; the total duration of pain medication is unknown.

#### 3.1.6. Follow-Up

None of the cats underwent a re-evaluation of cTnI. Only eight cats had serial echocardiographic examinations performed. Five cats were euthanized before full recovery was achieved: two of them were euthanized due to developing arterial thrombo-embolism (ATE), and three of them were euthanized due to financial restrictions after a few days of hospitalization. Since these cats had increased cTnI levels upon presentation, as well as an HCM phenotype, and had recently undergone general anesthesia, they were highly suspected of TMT and/or myocarditis.

There were no significant statistical differences between survivors and non-survivors of the TMT group regarding age (*p* = 0.951), weight (*p* = 0.426), and doses of medetomidine (*p* = 0.711), ketamine (*p* = 0.462), atipamezole (*p* = 0.195), or meloxicam (*p* = 0.371).

### 3.2. Control Group

The control group consisted of 180 males (60.0%) and 120 females (40.0%). A total of 15 different breeds were represented in the control group, of which the Domestic Shorthair was the most common breed (70.0%; 210/300). Occasional breeds were British Shorthair (8.0%; 24/300), Ragdoll (6.0%; 18/300), and Maine Coon (3.7%; 11/300), and mixed breeds included British Shorthair (7/300), Ragdoll (4/300), and Maine Coon (2/300). Furthermore, the breed distribution consisted of the Norwegian Forest Cat (5/300), Bengal (4/300), and Oriental Shorthair (3/300). Two cats were included from the Siamese, Persian, Sacred Birmans, Sphynx, and British Longhair breeds. One cat was included from the following breeds: Burmese, Blue Russian, and Scottish Fold. The median age was 202 days (126–715). The median body weight was 3.3 kg (1.88–6.45). All cats were placed on a heating pad while under anesthesia. All male cats and 20/120 (16.7%) females received oxygen flow-by. The remainder of the female cats (83.3%) were intubated, received oxygen and isoflurane via an endotracheal tube, and were monitored using capnography. The total duration of anesthesia is unknown. There were two cats (0.7%) with retained canine teeth, eight cats (2.7%) with an umbilical hernia, and seven cats (2.3%) with unilateral cryptorchidism. These abnormalities were treated during the same anesthesia. 

In the control group, all cats were anesthetized with ketamine (median 4.29 mg/kg; range 2.08–6.45) and an alpha-2 agonist, dexmedetomidine (median 25 µg/kg; range 10–51). All but one cat received atipamezole (median 0.12 mg/kg; range 0–0.39) after the procedure. Two hundred and ninety-nine cats (99.7%) received an anti-inflammatory drug during or shortly after the surgery; of those, 295 (98.7%) received meloxicam (median 0.29 mg/kg; range 0.18–0.38), and four (1.3%) received tolfenamic acid (median 4 mg/kg; range 4 mg/kg). Two hundred and seventy-nine cats (93.0%) also received buprenorphine (median 12.9 µg/kg; range 0.01–19) for additional pain management. Of the male cats, 135/180 (75.0%) received intratesticular lidocaine (median 1.08 mg/kg; range 0.64–1.75) divided equally over both testicles (in males without cryptorchidism). Eight (2.7%) cats did not receive buprenorphine or lidocaine, of which seven (87.5%) cats were male. Postoperatively, an NSAID was prescribed for the continuation of pain medication for a minimum of 3 days in 127 (42.3%) cats.

A total of 15 cats (5.0%) developed complications after surgery. Nine cats developed a wound infection, which was the most common complication. Three cats had post-surgery bleeding requiring either longer surgery in one case or a second surgery in two cases. Three cats had gastrointestinal signs; one cat had a wound infection and diarrhea. One cat developed an umbilical hernia post-surgery. None of the cats in the control group developed respiratory signs in the first weeks after anesthesia.

### 3.3. Comparison of Data

The two groups did not differ significantly regarding age (*p* = 0.085), weight (*p* = 0.985), and sex (*p* = 0.124). Breed distribution was significantly different (*p* = 0.015), with 70.0% Domestic Shorthair cats in the control group and 40% Domestic Shorthairs in the study group.

Since the cats in the study group mostly received medetomidine and the cats in the control group received dexmedetomidine, doses were adjusted for comparison as equipotent medetomidine doses. The medetomidine dose (*p* = 0.401) and antidote of alpha-2 agonists, atipamezole, did not differ significantly (*p* = 0.910) between the two groups.

The dose of ketamine was significantly higher in the TMT group than in the control group (*p* < 0.001), and the dose of meloxicam was significantly lower in the TMT group (*p* < 0.001). It is important to note that none of the TMT cats received buprenorphine or intratesticular lidocaine. See Table 2.

Fisher’s exact tests were undertaken for additional binary measures. These showed no significant difference for anesthetic monitoring (*p* = 0.060), anesthetic record-keeping (*p* = 0.569), and postoperative administration of NSAIDs (*p* = 0.193). Significant differences were found for lidocaine (*p* < 0.001), buprenorphine (*p* < 0.001), and oxygen supplementation (*p* < 0.001). See Table 3.

Results from the Firth binary logistic regression suggested that higher doses of ketamine were significantly associated with TMT (*p* = 0.014), as were lower doses of buprenorphine (0 = 0.030) and less oxygen supplementation (*p* = 0.017). 

## 4. Discussion

Although both groups consisted of young cats undergoing general anesthesia for gonadectomy in multiple first-opinion practices, the anesthetic protocols of the study group and control group differed more than initially anticipated.

Firstly, buprenorphine and intratesticular lidocaine were not used as additional analgesia in the study group. Ninety-three percent of cats in the control group (male and female) received buprenorphine, and 75% of male cats received a locoregional block with lidocaine. Both findings were significantly different between the study and control groups. Based on the Firth regression, low doses of buprenorphine are associated with the development of TMT. Low doses of lidocaine were not associated with TMT; this could be due to the low number of male cats with TMT in the study group.

Buprenorphine is the most commonly used opioid in feline analgesia [26,27,28]. Buprenorphine has a long-acting analgesic effect and has little effect on hemodynamics or the respiratory system [26,27,28,29]. This analgesic effect results in a lower feline pain score [26]. Low doses of buprenorphine may result in inadequate or insufficient perioperative analgesia. However, the absence of objective data to evaluate analgesic efficacy in either group may limit the ability to draw robust conclusions regarding this possibility.

Lidocaine is used as a local anesthetic and is recommended in anesthetic protocols for orchiectomy since it reduces the nociceptive response to surgery with intratesticular and subcutaneous injections [30]. No neurologic or cardiovascular effects are expected at the given doses (1.07 mg/kg), since plasma concentrations will be below toxic concentrations for cats [31]. An intratesticular block has been reported to improve intraoperative analgesia, which results in lower pain scores and, therefore, a lower stress response [26,32].

Potentially insufficient analgesia could result in an increased stress response, excessive release of catecholamines, and therefore, contribute to TMT [2,5,26]. Pain results in a stress response and sympathetic nervous system activity [26,33]. This activation stimulates endogenous catecholamine production [34]. Experimental evidence suggests that oxidative stress and oxidation of catecholamines may be involved in catecholamine-induced cardiomyopathy [35]. An increase in sympathomimetic activity results in an increased heart rate, stroke volume, and blood pressure, leading to increased myocardial oxygen demand [34,36,37,38]. Furthermore, increased catecholamine production causes oxidative damage to the heart by reducing coronary perfusion pressure during diastole [11,34,36]. This oxidative stress may result in myocardial damage, further provoking myocarditis due to ischemic damage [5,11,34]. The exact mechanisms of catecholamine cardiotoxicity are not fully understood [35]. As discussed above, the absence of objective data on analgesic efficacy precludes definitive conclusions. Therefore, this remains a hypothetical explanation.

Secondly, the doses of ketamine and meloxicam differed significantly. The dose of ketamine was significantly higher in the study group (*p* < 0.001). Ketamine is a dissociative anesthetic agent that affects several organ systems. Consequently, it may cause serious side effects [34,36,37]. Focusing on its cardiovascular effects, it increases the production of endogenous catecholamines, which may cause oxidative damage to the heart and may potentiate myocarditis and/or TMT [34,36]. The increase in myocardial oxygen demand is not compensated for by the increase in coronary blood flow caused by ketamine [34,36]. The release of catecholamines is dose-dependent, and thus, increasing the dose of ketamine may induce a cardiotoxic effect [34].

The meloxicam dose was significantly lower in the study group (*p* < 0.001). Meloxicam is a non-steroidal anti-inflammatory drug (NSAID). Non-steroidal anti-inflammatory drugs have anti-inflammatory, analgesic, and antipyretic effects; they inhibit cyclooxygenase enzymatic activity and limit prostaglandin production [39,40]. Some prostaglandins are produced after tissue damage and trigger inflammation, causing peripheral sensitization [38]. Feline myocarditis and TMT are forms of acute inflammation, including myocardial infiltration with inflammatory cells, which leads to cardiomyocyte damage [2,6,41,42]. The anti-inflammatory effect of NSAIDs is used to decrease pro-inflammatory processes in the myocardium in humans [42]. Additionally, meloxicam is an effective analgesic in cats and is approved for postoperative pain [40]. Pain itself may lead to stress, which may trigger myocarditis and/or TMT [2,5,26]. Following the Firth regression, lower meloxicam doses were not associated with the development of TMT.

Tolfenamic acid was administered to six cats in total: two female cats in the study group, three female cats, and one male cat in the control group. Tolfenamic acid is an NSAID, and based on one study in cats, it produces similar analgesic effects as meloxicam for ovariohysterectomy-related pain [43]. This group was not considered to have less analgesia than the cats who received meloxicam.

Importantly, postoperative NSAID administration did not differ significantly between the groups.

It is unclear to what extent inadequate analgesia and high doses of ketamine contribute simultaneously to the development of TMT, given that increasing ketamine doses are expected to produce greater dissociative–analgesic effects [34].

A limitation of the current study is the difference in alpha-2 agonist use between the two groups. Cats in the control group received dexmedetomidine, whereas cats in the study group received medetomidine. Although these agents can be compared for their pharmacological properties, the clinical relationship is not linear. However, a clinical comparison in the current study is difficult due to the lack of objective data regarding sedation depth, analgesic response, and perioperative cardiovascular effects. Therefore, the doses were compared based on their pharmacological receptor selectivity [44]. Dexmedetomidine is the dextrorotatory optical isomer of medetomidine and causes nearly all pharmacological effects of medetomidine at a 50% dose [44,45]. At this equipotent dose, dexmedetomidine causes comparable sedative, analgesic, and cardiorespiratory effects in cats, as well as similar muscle relaxation and recovery time [44,45,46]. However, as mentioned above, the 1:2 ratio for dexmedetomidine-to-medetomidine is not clinically linear, and there are known differences in the duration of sedative effects, dose–response characteristics, and cardiovascular effects [44,45]. Therefore, the conversion used should be interpreted with caution as equivalence is not considered for all clinically relevant effects [44,45].

At higher doses of dexmedetomidine (50–75 µg/kg), side effects such as reflex bradycardia are slightly more pronounced compared to the equipotent medetomidine dose [44]. Such high doses were not given to the cats in our study.

It was remarkable that 93.3% of cats in the study group received medetomidine, compared to 0% in the control group. Dexmedetomidine might have a potential cardioprotective role in humans undergoing cardiac surgeries [47,48]. Dexmedetomidine reduces myocardial ischemia as a result of its anti-inflammatory, antioxidant, and anti-apoptotic effects. It also has the potential to reduce cTnI levels [49,50]. No comparable studies were found for medetomidine, since this agent is not used in human medicine. Medetomidine has marked cardiovascular effects in cats, including bradycardia and hemodynamic effects [51]. No significant differences were found in the equipotent doses of the alpha-2 agonists between the groups. However, it might be possible that the use of dexmedetomidine versus medetomidine did play a role in the development of TMT.

Although 40.0% of cats in the control group were female and 60% of cats in the study group were female, this difference was not significant. There was no significant difference in age and body weight between the two groups. Higher analgesia doses might be expected in female cats since ovariohysterectomy requires more postoperative analgesia than orchiectomy [52]. It might be suggested that the cats from the study group experienced increased pain, as they received significantly lower doses of meloxicam and no buprenorphine or lidocaine. Recognizing and treating pain in cats is generally difficult [53]. Multimodal analgesia is preferred to maximize the analgesic effect [26,54,55]. Both an opioid and an NSAID are advised in cats undergoing ovariohysterectomy [56]. Optimal analgesia is achieved when buprenorphine is combined with an NSAID, locoregional anesthesia, or both [57]. Preventive analgesia limits the development of central and peripheral sensitization caused by surgery and reduces postoperative pain [26]. Since the difference between sexes is not significant, analgesia might have been less effective in female than in male cats.

There was a significant difference regarding breed disposition with fewer Domestic Shorthairs in the study group. However, the Domestic Shorthair was the most common breed in both groups, with 40.0% in the study group and 70.0% in the control group. There is no known breed predisposition for the development of TMT [1,2]. More studies are necessary to evaluate a possible breed predisposition, as this is true for other feline cardiomyopathies such as HCM [57].

Apart from the use of anesthetic and analgesic agents, other causes for the development of TMT following gonadectomy can be hypothesized [23].

First, Takotsubo cardiomyopathy should be considered; this is a catecholamine-induced cardiotoxicity following an acute stress response [5]. Several triggering stressors have been described, including both emotional and physical stress [5,58,59]. Emotional stressors include fear and anger, and physical stressors include acute medical illness, trauma, neurologic disease, and infection [5,58]. Perioperative stress represents a combination of both emotional and physical stressors [5,58]. Any stress reaction may result in acute sympathetic nervous system activation due to excessive catecholamine release, inducing myocardial injury and myocardial edema with reversible myocardial thickening [1,2,5,23,24]. Increased myocardial and circulating catecholamines lead to ventricular dysfunction through multiple mechanisms, including direct catecholamine-induced injury, oxidative stress, coronary microvascular dysfunction, and beta-adrenergic receptor overstimulation [5,60,61]. Takotsubo cardiomyopathy involves not only catecholaminergic but also ischemic mechanisms leading to myocarditis [5]. Travel to the veterinary clinic, perioperative handling, management and monitoring, as well as hospitalization and the surgery itself may cause severe stress, resulting in an excessive release of catecholamines.

Second, myocarditis and transient myocardial edema could be the result of sepsis or a systemic infection [2,10,23]. Notably, the cat in the case report by Wang et al. (2024) [23] received antibiotics; however, none of the cats in our study received antibiotics prior to clinical improvement. Sepsis was not present in our study population.

Finally, a delayed drug reaction cannot be excluded. In the case report by Wang et al. (2024) [23], the cat received several medications, including anesthetics, analgesics, and antibiotics that might induce TMT [1,23]. Several pharmacological agents have been associated with myocarditis in humans due to hypersensitive drug reactions [6,7,11]. It is plausible that all cats from the study group experienced a delayed drug reaction to one or more of the agents used.

Therefore, TMT can be associated with procedures under general anesthesia, either by inducing a perioperative stress response, systemic inflammation or infection, and/or delayed drug reactions [24].

The retrospective design resulted in the largest limitations of this study.

Firstly, the study group size was relatively small. A few reasons for this are (1) the low incidence of TMT [1,2]; (2) the fact that approximately one quarter of general practitioners had incomplete anesthetic records, thereby excluding these cats from the study; (3) financial limitations; and (4) a lower number of cats referred in the final years of the study due to increased knowledge of TMT by general practitioners. Financial restrictions had impacted both the initial hospitalization and follow-up of the cats, inducing selection bias. This potentially led to an underestimation of TMT in cats due to euthanasia before any treatment or work-up could be performed. Due to increased knowledge of TMT, fewer cats were referred in the acute phase of CHF, but more referrals were made for echocardiography at a later phase of the disease. Another potential selection bias exists, as only cooperative clinics that provided medication doses were included. The limited number of cases restricts the robustness of multivariable analyses. Therefore, any conclusions drawn from these analyses should be interpreted with caution.

Secondly, the criteria for diagnosing TMT as defined by Romito et al. (2023) [2] and Novo Matos et al. (2018) [1] were not completely met. A potential detection bias may have occurred; not all cats underwent serial echocardiography until normalization was noted, and none had repeated cTnI concentration measurements. Five cats were euthanized before resolution of TMT could be achieved; however, considering they were all young cats with recent (<2 weeks) general anesthesia, presenting with an HCM phenotype and very high cTnI values, TMT and/or feline myocarditis were suspected. Recovery in these cats could have been achieved with longer or more intensive hospitalization, as two of them were euthanized due to an ATE, and three due to financial restrictions regarding hospitalization. Echocardiographic follow-up was only performed in a handful of cats due to the owner’s financial concerns. In the early years of the study period, measuring cTnI was rare, and cTnI was not reassessed once a diagnosis was made. Normalization of cTnI over time can further confirm the diagnosis of TMT [2].

Usually, cardiac troponin concentrations in primary cardiac disease, such as HCM, only mildly increase (cTnI < 1 ng/mL) [3]. If those cats develop severe CHF, cTnI rarely increases above 1–2 ng/mL [1,3]. The marked elevation in cTnI is highly sensitive and specific for cardiac myocyte injury [3]. It is suggestive of acute myocarditis and thus also for TMT [1,2,3,11,37,40].

Generally, the diagnosis of TMT is challenging due to its apparent similarities to HCM [1,2]. The main differences at presentation between HCM and TMT are a younger age, the presence of antecedent events, and markedly elevated cTnI levels in cats diagnosed with TMT [1]. The echocardiographic changes are less pronounced in cats with TMT, the left ventricular walls are thinner, and the left atrium is smaller [1,2]. Treatment of TMT is similar to HCM in the acute phase and is symptomatic with diuretics [1,2]. The occurrence of an ATE with TMT is rare and requires intensive therapy [2,62].

Furthermore, none of the cats underwent invasive methods to confirm diagnosis, such as endomyocardial biopsy or cardiac magnetic resonance, which is necessary for a definitive diagnosis [1,2,6].

Preoperative cardio-specific exams were not performed in the study or in the control group. In first-opinion practice, it is uncommon to perform echocardiography in ASA I animals for gonadectomy. Therefore, occult cardiac diseases could have been undiagnosed and postoperative cardiac changes could have been present preoperatively.

Since the exact etiopathogenesis of TMT is hypothesized to be multifactorial, several triggers could cause the development of TMT in a predisposed cat. Not all of these concurrent factors could be excluded, which is another limitation of the current study.

Serological testing for *Bartonella henselae*, *Toxoplasma gondii*, or feline immunodeficiency virus was not performed. All of these pathogens have been associated with the development of TMT [12,13,14,17]. Despite the cats being considered healthy prior to anesthesia, these conditions could have been present without being clinically relevant at the time [12]. Perioperative stress, general anesthesia, and surgery could potentially be enough for these conditions to result in clinical illness.

Surgery-related contributors that could not be excluded are surgical experience and technique, tissue handling and trauma, operation and anesthetic duration, and intraoperative stress. These contributors were not controlled and could lead to the development of TMT. The cats originated from multiple general practices, inducing heterogeneity in surgery and anesthetic protocols. These surgical variables could unfortunately not be fully characterized for both groups due to the retrospective nature and incomplete patient records.

Additionally, the median time to development of clinical signs was 5 days, with a range from 1 to 14 days after the procedure. Another stressful event could have occurred between the surgery and the onset of clinical signs, such as inadequate postoperative pain medication, and wearing a cone or medical pet shirt, thus limiting the cat’s movements.

Another weakness of this study is the lack of standardization of anesthetic protocols. The two groups were not operated on at the same practices or by the same practitioners, inducing bias in the operating circumstances. Several uncontrolled confounders include preoperative and postoperative stress-handling, monitoring during anesthesia, experience of practitioners, duration of anesthesia, anesthetic depth, postoperative duration of pain medication, and other possible postoperative stress factors. Unfortunately, it was not possible to obtain records of the same referring practices that performed the surgeries in the study group due to privacy issues. To ensure that a similar source population was used for the control group, the authors chose other referring general practices in the same geographic region. A high case–control ratio (1:20) was used to improve precision due to the limited number of cases in the study group. Controls were selected from multiple veterinarians and clinics within the same source population to reduce potential practitioner-related effects, such as cat-handling, the chosen anesthetic protocol, monitoring opportunities, operating experience, and postoperative care. It is unknown whether cat-friendly handling occurred for the cats in the study group during the procedure.

Information bias is possible due to incomplete anesthetic records in the study group, with missing documentation of perioperative variables such as oxygen supplementation and capnography. Therefore, anesthetic events such as apnea, hypoxemia, or even cardiac arrest could have happened but were not recorded. Following Firth regression, a lack of oxygen supplementation was associated with the development of TMT. The absence of oxygen supplementation might result in hypoxemia. Ischemic myocardial damage has been described following hypoxemia during general anesthesia [63]. This ischemic damage could result in acute myocarditis or TC [5]. Additionally, it is unknown whether the anesthetic protocols were sufficient regarding sedation, anesthesia, and analgesia. In both groups, there is no information regarding sedation depth, analgesic response, cardiovascular responses, or perioperative stress indicators.

Information bias may have occurred due to misclassification of the control health status. The control group was considered healthy based on a physical examination and considered ASA I prior to the procedure. None of the cats in the control group underwent additional testing, such as echocardiography or blood examination. This does not exclude any occult cardiac disease, such as asymptomatic myocarditis postoperatively, which occurs in humans [41]. Myocardial damage does not always result in congestive heart failure. This might exclude the control group completely and distort the statistical analysis. However, none of the cats in the control group developed myocardial damage or myocarditis that resulted in congestive heart failure. Therefore, the cats in the control group can be considered representative.

Human patients with acute myocarditis have a good prognosis [41], compatible with the excellent prognosis of TMT in cats [1,2]. In our study, only 10/15 cats survived. Yet, of the five non-survivors, three were euthanized due to financial restrictions and two were euthanized due to an ATE, which might have resolved with a longer and more intensive therapy. Additionally, no significant differences were found between the anesthetic protocols of survivors and those of non-survivors. 

Further studies are warranted to evaluate the effect of anesthetic protocols.

## 5. Conclusions

The small study group size and lack of standardization in anesthetic protocols limit the ability to draw robust conclusions regarding drug effects in the development of TMT. However, significant differences between the doses of ketamine and meloxicam combined with the differences in use of buprenorphine and lidocaine may suggest using multimodal analgesia to ensure adequate pain relief. Inadequate analgesia and lack of oxygen supplementation might be associated with the development of feline myocarditis and TMT.

## Figures and Tables

**Table 1:** Days until respiratory signs developed, cardiac troponin (cTnI) concentrations, ketamine and non-steroidal anti-inflammatory drugs (NSAIDs), echocardiographic findings with left atrium/aorta ratio (LA/Ao), left ventricular wall posterior wall thickness end-diastole (LVWPD), and survival (>15 months) for cats belonging to the transient myocardial thickening group.

Cat	Days Until Clinical Signs	cTnI (ng/mL)	Ketamine (mg/kg)	NSAID		Echocardiographic Findings		Survival (>15 Months)
				Meloxicam (mg/kg)	Tolfenamic Acid (mg/kg)	LA/Ao	LVWPD (mm)	
1	3	18.21	6.06	0.2	0	1.95	6.81	No, ATE
2	1	16.98	6.78	0	4	2.11	6.4	No
3	14	9.09	7.21	0.2	0	1.82	6.93	Yes
4	7	2.66	6.94	0	0	2.56	9.3	Yes
5	2	2.06	4.52	0.2	0	1.58	4.8	Yes
6	3	22.89	10.34	0.26	0	1.45	5.26	Yes
7	10	47.38	4.8	0	4	1.93	6.4	No
8	5	24.47	4.55	0.23	0	2.28	6.9	No
9	14	5.51	7.14	0.2	0	1.72	8	Yes
10	9	2.73	4.56	0.18	0	1.19	6.57	Yes
11	9	4.41	4.65	0.23	0	1.92	6.93	Yes
12	14	14.88	7.14	0	0	2.03	6.1	No, ATE
13	3	2.48	8	0	0	1.98	6.27	Yes
14	10	1	6.97	0.3	0	1.7	8	Yes
15	14	12.15	4.15	0.2	0	1.8	6.5	Yes

**Table 2:** Median (range) doses of drugs used in the anesthetic protocols of cats that developed TMT and those who did not. (^1^) Equipotent dose of dexmedetomidine.

Anesthetic Drugs	Study Group	Control Group	*p*-Value
Ketamine (mg/kg)	6.78 (4.52–10.35)	4.29 (2.08–6.45)	<0.001
Medetomidine (µg/kg)	70 (10–100)	50 (20–102) ^1^	0.401
Atipamezole (mg/kg)	0.15 (0–0.26)	0.12 (0–0.39)	0.910
Meloxicam (mg/kg)	0.2 (0–0.3)	0.29 (0–0.38)	<0.001
Tolfenamic acid (mg/kg)	4 (4)	4 (4)	
Buprenorphine (µg/kg)	0	12.9 (0–19)	
Lidocaine (mg/kg)	0	1.08 (0–1.75)	

**Table 3:** Additional variables such as buprenorphine, lidocaine, oxygen supplementation, anesthetic record-keeping, anesthetic monitoring, and postoperative non-steroid anti-inflammatory drug (NSAID) administration used in the study and control groups.

Additional Variables	Study Group	Control Group	Fisher Test *p*-Value
Buprenorphine (Yes/No)	0%	93%	<0.001
Lidocaine (Yes/No)	0%	45%	<0.001
Oxygen supplementation	33%	100%	<0.001
Anesthetic record-keeping	80%	71%	0.569
Anesthetic monitoring	13%	38%	0.060
Postoperative NSAID	60%	42%	0.193

## Data Availability

The data that support the findings of this study are available from the corresponding author upon reasonable request.

## Abbreviations

The following abbreviations are used in this manuscript:

ASA I	American Society of Anesthesiologists I
ATE	Arterial thrombo-embolism
CHF	Congestive heart failure
cTnI	Cardiac Troponin I
HCM	Hypertrophic cardiomyopathy
LVWAT	Left ventricular wall thickness
NSAID	Non-steroidal anti-inflammatory drug
TC	Takotsubo cardiomyopathy
TMT	Transient myocardial thickening
